# Preservation of the articular capsule and short lateral rotator in direct anterior approach to total hip arthroplasty

**DOI:** 10.1007/s00590-018-2166-2

**Published:** 2018-03-09

**Authors:** Akio Kanda, Kazuo Kaneko, Osamu Obayashi, Atsuhiko Mogami, Itaru Morohashi

**Affiliations:** 1 Department of Orthopaedic Surgery, Juntendo Shizuoka Hospital, Izunagaoka 1129, Izunokuni-Country, Shizuoka 410-2295 Japan; 2 Department of Orthopaedic Surgery, Juntendo University, Hongou 3-1-3, Bunkyou Ward, Tokyo, 113-8431 Japan

**Keywords:** Total hip arthroplasty, Direct anterior approach, The internal obturator muscle belly with the conjoint tendon, The posterosuperior articular capsule, Dislocation

## Abstract

**Introduction:**

In total hip arthroplasty via a direct anterior approach, the femur must be elevated at the time of femoral implant placement. For adequate elevation, division of the posterior soft tissues is necessary. However, if we damage and separate the posterior muscle tissue, we lose the benefits of the intermuscular approach. Furthermore, damage to the posterior soft tissue can result in posterior dislocation. We investigate that protecting the posterior soft tissue increases the joint stability in the early postoperative period and results in a lower dislocation rate.

**Methods:**

We evaluated muscle strength recovery by measuring the maximum width of the internal obturator muscle on CT images (GE-Healthcare Discovery CT 750HD). We compared the maximum width of the muscle belly preoperatively versus 10 days and 6 months postoperatively. As clinical evaluations, we also investigated the range of motion of the hip joint, hip joint function based on the Japanese Orthopaedic Association hip score (JOA score), and the dislocation rate 6 months after surgery.

**Results:**

The width of the internal obturator muscle increased significantly from 15.1 ± 3.1 mm before surgery to 16.4 ± 2.8 mm 6 months after surgery. The JOA score improved significantly from 50.8 ± 15.1 points to 95.6 ± 7.6 points. No dislocations occurred in this study.

**Conclusions:**

We cut only the posterosuperior articular capsule and protected the internal obturator muscle to preserve muscle strength. We repaired the entire posterosuperior and anterior articular capsule. These treatments increase joint stability in the early postoperative period, thus reducing the dislocation rate.

**Level of evidence:**

Therapeutic, Level IV.

## Introduction

The anterior approach for total hip arthroplasty was first described in the German literature by Hueter [1] in 1883; Smith-Petersen [2] described the anterior approach for mold arthroplasty in the English literature in 1949. The anterior approach described by Judet in 1947 was a modified Smith-Petersen approach, following the principles of minimally invasive surgery [3]. In 1980, Keggi used the term ‘direct anterior approach’ to describe minimally invasive surgery via an anterior approach [4]. This approach is intermuscular and internervous, and many studies have shown that it allows early recovery of muscle strength and improved walking ability in the early postoperative period [5–7]. Sariali hypothesized that these good results are achieved with an anterior approach because it requires no cutting of muscles or tendons [8]. However, there is a steep learning curve for the surgical procedure; perioperative and postoperative complications are more frequent without correct surgical technique [6, 9]. The surgical technique on the femoral side is particularly difficult, namely elevation of the femur to place the femoral implant. To adequately elevate the femur, it is necessary to divide the posterior soft tissues, particularly the articular capsule of the hip joint. However, if we damage and separate the posterior muscle tissue, we lose the benefits of the intermuscular approach. Furthermore, damage to the posterior muscle tissue and articular capsule can result in posterior dislocation, which is an important complication of total hip arthroplasty [10]. Barton postulated that inherent stability remains, because muscles are not detached posteriorly or anteriorly. However, if care is not taken to avoid damage to the posterior structures, dislocations will inevitably occur [11]. Moreover, Ito reported that it is important to preserve the short external rotators to ensure postoperative hip stability, as has been documented for other approaches such as the posterior approach [12]. Thomas described advancements in surgical techniques, including soft tissue repair, that may further reduce the risk of dislocation [13]. Therefore, we have devised surgical techniques for posterior muscle tissue preservation. Specifically, we only cut the posterior articular capsule in a proximal–distal direction at the midpoint of the attachment site on the femur and the attachment site on the posterior acetabular roof. Moreover, in various approach, the dislocation rate can be reduced through joint capsule reconstruction [9, 14–17]. Thus, we repair the posterior articular capsule with absorbable suture after femoral implant placement. Moreover, we close the anterior articular capsule, completely repairing the articular capsule to decrease the risk of hip dislocation. With these surgical techniques, we aim to prevent early dislocation and to promote early muscle strength recovery. Imaging methods, such as a computed tomography (CT) and magnetic resonance imaging (MRI), are considered the most accurate methods for in vivo quantification of body composition on the tissue level, and skeletal muscle can be compartmentalized into individual muscle. This level of specificity in tissue composition is only possible with CT or MRI [18]. In other report, skeletal muscle measurements using MRI are reproducible and correlate closely with CT [19]. When we use the CT, patients are exposed to radiation, but when we use the MRI, patients are not. Therefore, the MRI is more safety for patients and it should be used for a patient. However, we performed the CT of the same part to observe an osseous state in all patients. Therefore, in this study, we evaluated muscle strength recovery by examining CT images of the internal obturator muscle and comparing the maximum muscle belly size on the affected versus unaffected side. Because of the very proximal attachment site of the rotator muscles, the femoral attachment site of the internal obturator muscle is most easily damaged during elevation of the femur [10]. Therefore, if there is little soft tissue injury to the posterior element, we can evaluate recovery of this muscle as an indicator of early recovery of muscle strength and improved walking ability in the early postoperative period and of joint stability, especially in the posterior direction.

## Methods

### Patients

This study included patients with a diagnosis of osteoarthritis or avascular necrosis of the femoral head who underwent primary total hip arthroplasty at our hospital between March 2014 and March 2016. All procedures were performed by the same surgeon via a direct anterior approach and all patients had 6 months of postoperative follow-up. We performed 103 total hip arthroplasty procedures during this period; 16 patients (two men, 14 women) were lost to follow-up. The study cohort thus comprised 87 patients (11 men, 76 women), with a mean age of 65.6 ± 11.1 years (Table 1).

**Table 1:** Patient demographics, operative time and blood loss

Number of patients	87
Age (years) (SD)	65.6 ± 11.1 years
Sex (no.)	11 males, 76 females
Operative mean time (min)	126.0
Mean amount of the lost blood (g)	426.9

### Study design

**Fig. 1:**
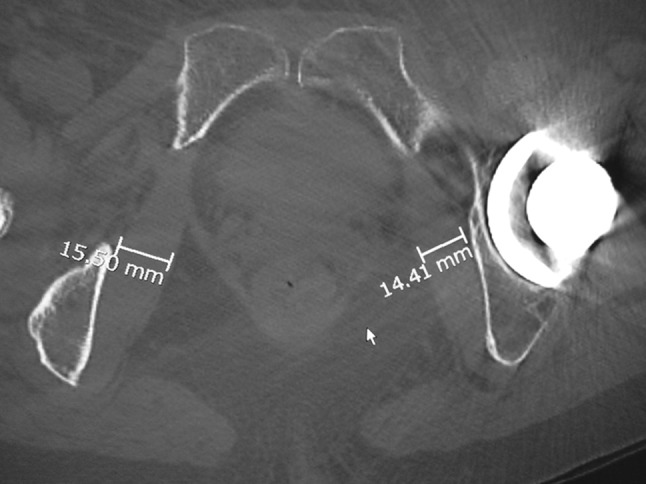
We calculated the maximum width of the internal obturator muscle belly on CT images of the affected and unaffected sides preoperatively, 10 days postoperatively, and 6 months postoperatively

This was a retrospective study. We evaluated muscle strength recovery by measuring the maximum width of the internal obturator muscle belly with the conjoint tendon on CT images (GE-Healthcare Discovery CT 750HD) (Fig. 1). We compared the maximum muscle belly width on the affected versus unaffected sides preoperatively. We also compared the maximum on the affected and unaffected side before surgery versus on the affected side 10 days and 6 months postoperatively. We performed the muscle measurements with image processing software (Nazca; Astro-Stage, Tokyo). For clinical evaluation, we investigated the range of motion of the hip joint, hip function based on the Japanese Orthopaedic Association hip score, and dislocation rate 6 months after surgery.

### Implants

Seventy-seven patients received an uncemented porous cup (Trident PSL cup; Stryker Orthopaedics, Mahwah, New Jersey, USA) with a highly crosslinked Trident X3 polyethylene liner (Stryker Orthopaedics). Twenty-three of the 77 patients with the Trident cups received uncemented, double-tapered, fully hydroxyapatite-coated femoral stems (Accolade II or Accolade I; Stryker Orthopaedics) with a V40 alumina ceramic head (Stryker Orthopaedics) and a 32-mm articulation. Fifty-four of the 77 patients with Trident cups received Optimys short-stem femoral implants (Mathys Ltd., Bettlach, Switzerland) with a Bionit2 alumina ceramic head (Mathys Ltd.) and a 32-mm articulation. The remaining 10 patients received a Kyosera Sqrum shell (Kyocera Medical Materials, Osaka, Japan) with an uncemented, double-tapered, fully hydroxyapatite-coated stem (Kyosera J Taper; Kyocera Medical Materials) and a 910 zirconia head (Kyocera Medical Materials) with a 32-mm articulation.

### Approach

**Fig. 2:**
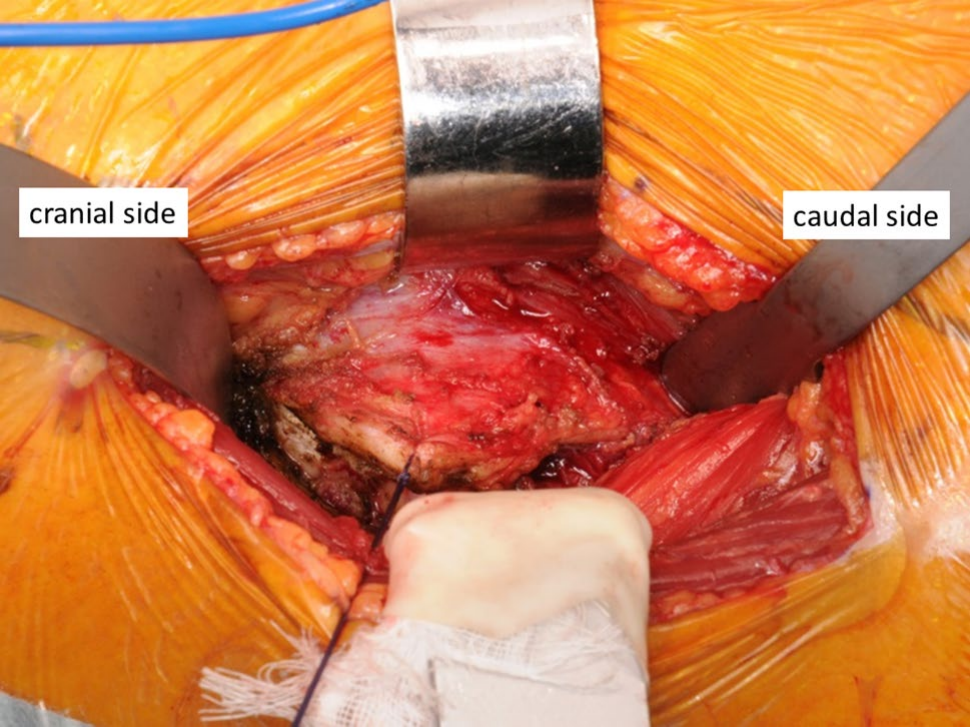
We incised the anterior articular capsule at its attachment to the femur and extended the incision proximally and then lifted the capsule like a curtain

**Fig. 3:**
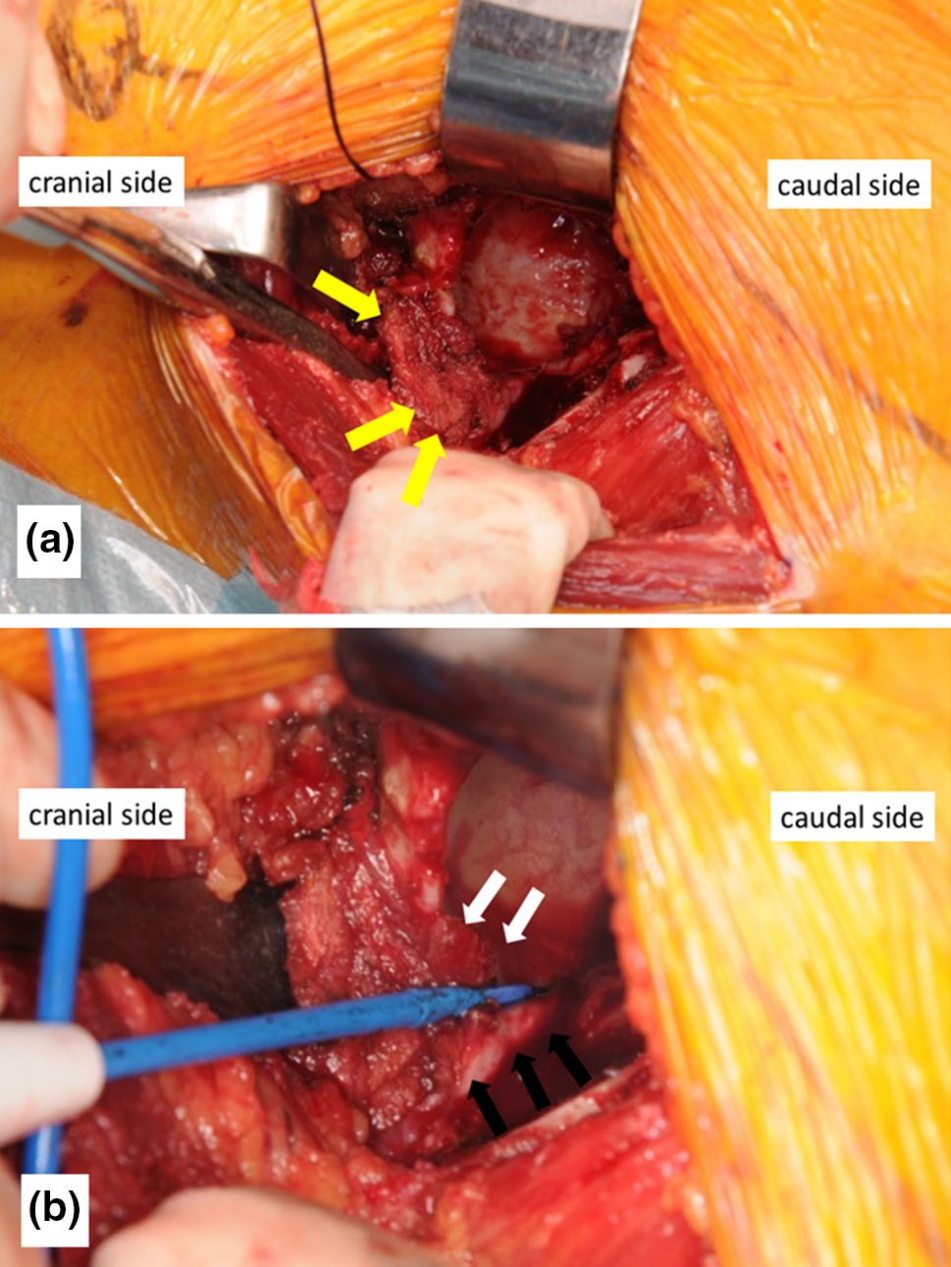
**a** We separated the posterior articular capsule from the piriform muscle and rotator muscles, including the internal obturator muscle. The arrows show the posterosuperior articular capsule. **b** We cut the posterosuperior articular capsule in a proximal–distal direction at the midpoint of the femoral attachment site and at the attachment site of the posterior acetabular roof. The black arrows show the attachment site on the femur. The white arrows show the attachment site on the posterior acetabular roof

**Fig. 4:**
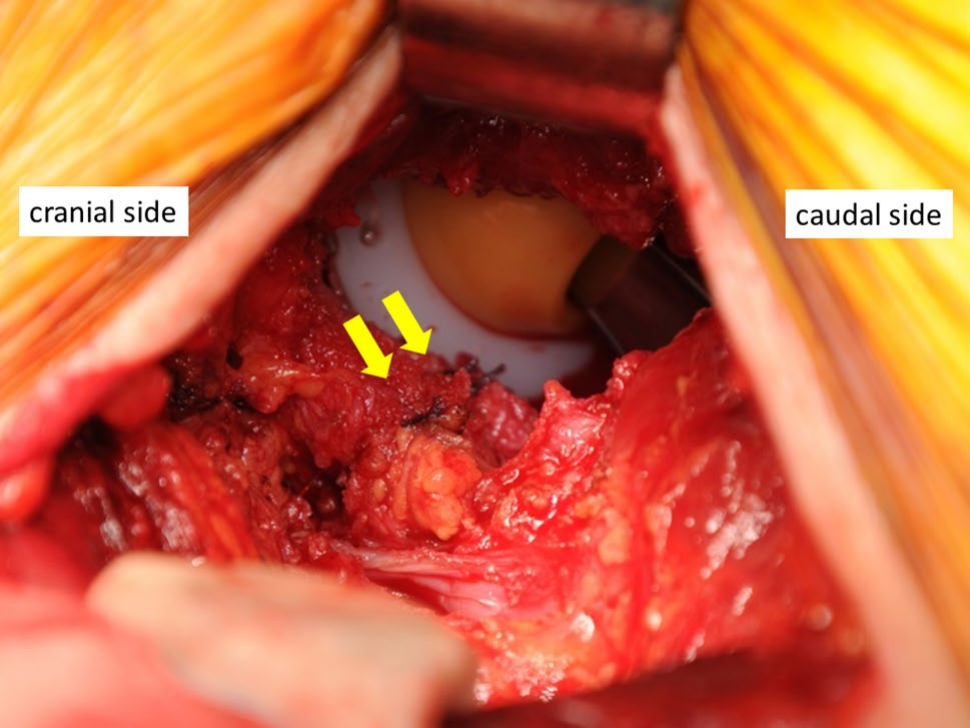
We closed the posterior articular capsule with absorbable suture. The arrows show the suture

**Fig. 5:**
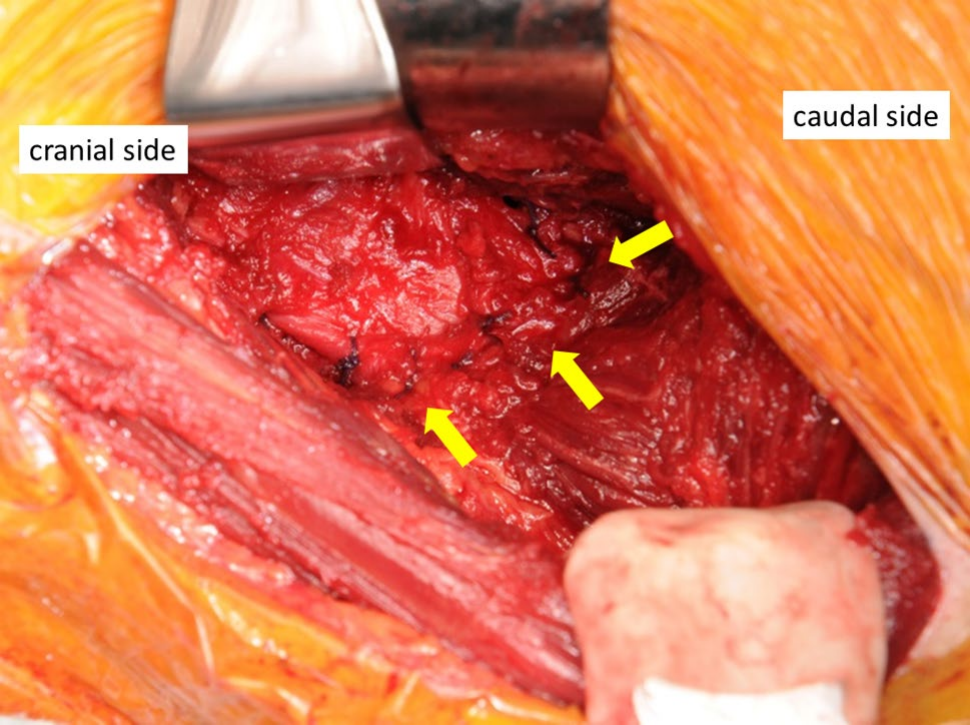
We closed the anterior articular capsule. The arrows show the absorbable suture

All procedures were performed via a modified Smith-Petersen approach as described by Nogler et al. [20]. The mean operation time was 126 min, and the mean intraoperative blood loss was 426 mL. Skin incisions were approximately 10 cm in length. This surgical approach used the space between the tensor fascia lata and the sartorius muscle, which is an internervous plane. Subsequently, we separated the rectus femoris and iliopsoas muscles from the articular capsule. We incised the anterior articular capsule at its attachment site on the femur and extended the incision proximally and then lifted the capsule like a curtain (Fig. 2). After removing the femoral head, we separated the posterosuperior articular capsule from the piriform muscle and rotator muscles, including the internal obturator muscle (Fig. 3a). While protecting the muscle layer, we cut the posterosuperior articular capsule in a proximal–distal direction only at the midpoint of the femoral attachment site and the attachment site of the posterior acetabular roof (Fig. 3b). After placement of the femoral implant, we closed the posterior articular capsule with absorbable suture (Fig. 4). Finally, we closed the anterior articular capsule, achieving complete repair of the articular capsule (Fig. 5). With these surgical techniques, we aim to prevent early dislocation and to promote early recovery of muscle strength.

### Outcome measures

- We compared the maximum muscle belly width on the affected versus unaffected sides preoperatively. We also compared the maximum on the affected and unaffected side before surgery versus on the affected side 10 days and 6 months postoperatively. CT scans were acquired in 0.625-mm-thick slices, and images were reconstructed in the axial plane with 0.625 mm slice thickness. We performed measurements with image processing software (Nazca; Astro-Stage, Tokyo) (Fig. 1).
- For clinical evaluation, we investigated the range of motion of the hip joint, hip function based on the Japanese Orthopaedic Association hip score, and dislocation rate 6 months after surgery.

### Statistics

Statistical calculations were performed with ystat2000.xls software. The Wilcoxon *t* test was used to evaluate the range of motion of the hip joint and functional test scores. Repeated measures ANOVA and the Student–Newman–Keuls test were used to evaluate the maximum internal obturator muscle belly width on CT images.

## Results

1. There was a significant increase in the width of the internal obturator muscle belly, from 15.1 ± 3.1 mm before surgery to 16.4 ± 2.8 mm 6 months after surgery (Table 2).

**Table 2:** Maximum internal obturator muscle belly width

Preoperative affected side (SD)	Preoperative unaffected side (SD)	Affected side at postoperative 10 days (SD)	Affected side at postoperative 6 months (SD)
15.9 ± 3.1 mm	16.6 ± 3.7 mm *P < 0.01	15.8 ± 2.7 mm *P < 0.05	16.4 ± 2.8 mm *P < 0.01

*Repeated measures ANOVA, Student–Newman–Keuls test
2. The Japanese Orthopaedic Association hip score improved significantly from 50.8 ± 15.1 points before surgery to 95.6 ± 7.6 points 6 months after surgery (Table 3). No dislocations occurred in this study.

**Table 3:** The Japanese Orthopaedic Association hip score

	Preoperative	Postoperative 6 months	*P* value*
JOA score (SD)	50.8 ± 15.1 points	95.6 ± 7.6 points	*P* < 0.001

*Wilcoxon *t* test

## Discussion

The direct anterior approach is intermuscular and internervous. Many studies have shown that this approach allows early recovery of muscle strength and improved walking ability in the early postoperative period [5–7]. Sariali postulated that an anterior approach achieves these results because it does not require cutting any muscles or tendons [8]. This surgical procedure allows an intermuscular approach without muscle damage. However, this procedure has a steep learning curve [6] and an increased risk of perioperative and postoperative complications unless it is performed with correct surgical technique [9]. The surgical technique on the femoral side is particularly difficult, especially femoral elevation for implant placement. To achieve adequate elevation of the femur, division of the posterior soft tissues is necessary, particularly the articular capsule of the hip joint. However, if we damage and separate the posterior muscle tissue, we lose the benefits of the intermuscular approach. Furthermore, damage to the posterior muscle tissue and articular capsule can result in posterior dislocation, which is an important complication of total hip arthroplasty [10]. Ito et al. reported that the internal obturator muscle, the superior gemellus muscle, and the inferior gemellus muscle form a conjoined tendon and that the insertion of the conjoined tendon extends to the anterosuperior aspect of the greater trochanter, and moreover Ito stated that when we open the posterosuperior articular capsule to achieve implant insertion, the internal obturator muscle tends to be most damaged [12]. In another study, Meneghini reported that the piriform muscle or the conjoint tendon is damaged in 50% of patients when the femur is elevated for implant placement [10]. In a cadaver study, Matsuura reported that elevation of the femur is enabled when we open the posterosuperior articular capsule, but that the degree of elevation does not change even with release of the internal obturator muscle [21]. To preserve muscle strength, it is important to cut only the posterosuperior articular capsule during elevation of the femur, and it is necessary to protect the conjoint tendon including the internal obturator muscle. Barton postulated that inherent stability is maintained because muscles are not detached posteriorly or anteriorly. However, if care is not taken to protect posterior structures, dislocations will inevitably occur [11]. Moreover, Ito reported that it is important to preserve the short external rotators to ensure postoperative hip stability, as has been documented for other approaches such as the posterior approach [12]. Thomas reported advancements in surgical techniques, including soft tissue repair, that further reduced the risk of dislocation [13]. Therefore, it is important to protect the internal obturator muscle when cutting the articular capsule. In our study, the width of the internal obturator muscle belly increased significantly, from 15.1 ± 3.1 mm before surgery to 16.4 ± 2.8 mm 6 months after surgery. Because muscle atrophy resulting from damage during surgery is extremely obvious, this finding shows that there was no perioperative injury. Moreover, the Japanese Orthopaedic Association hip score improved significantly from 50.8 ± 15.1 points before surgery to 95.6 ± 7.6 points 6 months after surgery (Table 3). No dislocations occurred in this study. These findings indicate muscular strength recovery during the early postoperative period. However, even if we preserve the internal obturator muscle, dislocation remains a potential complication of total hip replacement. The dislocation rate is generally low with a direct anterior approach compared with other approaches [8, 11, 13, 22, 23]. However, most studies have reported some cases of dislocation. One recent study reported that there was no difference in the dislocation rate between patients undergoing total hip arthroplasty via a direct anterior approach versus a modern posterior approach [24]. Shang-Ju reported that the early postoperative hip-dislocation rate among patients who did not undergo posterior capsular repair was substantially higher than that among patients who underwent posterior capsular repair [14]. Prietzel et al. reported that Preservation and repair of the hip joint capsule causes an 88% reduction in the dislocation rate in primary THA in this large series including 1972 cases, operated via the Bauer or the anterolateral approach [15]. Moreover, White described formal repair of the posterior capsule and short external rotator tendons as a surgical approach to reduce the incidence of posterior dislocation after a posterolateral surgical approach for primary total hip replacement [16]. Thus, in various approaches, the dislocation rate can be reduced through joint capsule reconstruction. When we perform total hip arthroplasty via a direct anterior approach, it is necessary to repair the entire posterosuperior articular capsule, which we incise to elevate the femur, and to repair the anterior articular capsule, which we incise to access the hip joint. Because this procedure increases joint stability in the early postoperative period, the dislocation rate is reduced.

Limitations of this study are the small number of patients and possible errors in measurement of internal obturator muscle width. Also, this was a retrospective study with no control group.

In conclusion, in this study we incised only the posterosuperior articular capsule for femur elevation, maintaining the conjoint tendon including the internal obturator muscle to preserve muscle strength. We also repaired the entire posterosuperior articular capsule, which was incised to allow elevation of the femur, and we repaired the anterior articular capsule, which was incised to access the hip joint. The increased joint stability in the early postoperative period achieved with this treatment reduces the dislocation rate. When we performed total hip arthroplasty via a direct anterior approach without posterior soft tissue injury, patients achieved early recovery without dislocation.

## References

[1] Funfte Abteilung HC, Hueter C (1883). die Verletzung und Krankheiten des Huftgelenkes, neun und Zwanzigstes capitel. Grundriss der Chiruugie.

[2] Smith-Petersen MN (1949). Approach to and exposure of the hip joint for mold arthroplasty. J Bone Joint Surg Am.

[3] Judet J, Judet R (1959). The use of an artificial femoral head for arthroplasty of the hip joint. J Bone Joint Surg Br.

[4] Light TR, Keggi KJ (1980) Anterior approach to hip arthroplasty. Clin Orthop Relat Res (152):255-60 7438611

[5] Connolly KP, Kamath AF (2016). Direct anterior total hip arthroplasty: comparative outcomes and contemporary results. World J Orthop.

[6] Post ZD, Orozco F, Diaz-Ledezma C, Hozack WJ, Ong A (2014). Direct anterior approach for total hip arthroplasty: indications, technique, and results. J Am Acad Orthop Surg.

[7] Moskal JT (2011). Anterior approach in THA improves outcomes: affirms. Orthopedics.

[8] Sariali E, Leonard P, Mamoudy P (2008). Dislocation after total hip arthroplasty using Hueter anterior approach. J Arthroplasty.

[9] Lee GC, Marconi D (2015). Complications following direct anterior hip procedures: costs to both patients and surgeons. J Arthroplasty.

[10] Meneghini RM, Pagnano MW, Trousdale RT, Hozack WJ (2006). Muscle damage during MIS total hip arthroplasty: Smith-Petersen versus posterior approach. Clin Orthop Relat Res.

[11] Barton C, Kim PR (2009). Complications of the direct anterior approach for total hip arthroplasty. Orthop Clin North Am.

[12] Ito Y, Matsushita I, Watanabe I, Kimura T (2012). Anatomic mapping of short external rotators shows the limit of their preservation during total hip arthroplasty. Clin Orthop Relat Res.

[13] De Geest T, Fennema P, Lenaerts G, De Loore G (2015). Adverse effects associated with the direct anterior approach for total hip arthroplasty: a Bayesian meta-analysis. Arch Orthop Trauma Surg.

[14] Tsai SJ, Wang CT, Jiang CC (2008). The effect of posterior capsule repair upon post-operative hip dislocation following primary total hip arthroplasty. BMC Musculoskelet Disord.

[15] Prietzel T, Hammer N, Schleifenbaum S, Adler D (2014). The impact of capsular repair on the dislocation rate after primary total hip arthroplasty: a retrospective analysis of 1972 cases [Article in German]. Z Orthop Unfall.

[16] White RE, Forness TJ, Allman JK, Junick DW (2001). Effect of posterior capsular repair on early dislocation in primary total hip replacement. Clin Orthop Relat Res.

[17] Kwon MS, Kuskowski M, Mulhall KJ (2006). Does surgical approach affect total hip arthroplasty dislocation rates?. Clin Orthop Relat Res.

[18] Fosbøl MØ, Zerahn B (2015). Contemporary methods of body composition measurement. Clin Physiol Funct Imaging.

[19] Sinelnikov A, Qu C, Fetzer DT, Pelletier JS, Dunn MA, Tsung A, Furlan A (2016). Measurement of skeletal muscle area: comparison of CT and MR imaging. Eur J Radiol.

[20] Nogler M, Krismer M, Hozack WJ, Merritt P, Rachbauer F, Mayr E (2006). A double offset broach handle for preparation of the femoral cavity in minimally invasive direct anterior total hip arthroplasty. J Arthroplasty.

[21] Matsuura M, Ohashi H, Okamoto Y, Inori F, Okajima Y (2010). Elevation of the femur in THA through a direct anterior approach: cadaver and clinical studies. Clin Orthop Relat Res.

[22] Siguier T, Siguier M, Brumpt B (2004). Mini-incision anterior approach does not increase dislocation rate: a study of 1037 total hip replacements. Clin Orthop Relat Res.

[23] Matta JM, Shahrdar C, Ferguson T (2005). Single-incision anterior approach for total hip arthroplasty on an orthopaedic table. Clin Orthop Relat Res.

[24] Maratt JD, Gagnier JJ, Butler PD, Hallstrom BR, Urquhart AG, Roberts KCJ (2016). No difference in dislocation seen in anterior vs posterior approach total hip arthroplasty. J Arthroplasty.

